# Extra-medullary Plasmacytomas: A Case Series of Rare Hematological Tumors

**DOI:** 10.7759/cureus.73519

**Published:** 2024-11-12

**Authors:** Omar Halloumi, Fatima Safini, Radia Chakiri, Mohamed Mehdi El Fakiri, Salma Fares

**Affiliations:** 1 Department of Hematology, Agadir Faculty of Medicine and Pharmacy, Agadir, MAR; 2 Department of Radiation Therapy, Souss-Massa University Hospital, Agadir, MAR; 3 Department of Dermatology, Souss-Massa University Hospital, Agadir, MAR; 4 Department of Otorhinolaryngology, Souss-Massa University Hospital, Agadir, MAR

**Keywords:** cutaneous plasmacytoma, extra-medullary plasmacytoma, nasal plasmacytoma, ovarian plasmacytoma, solitary plasmacytoma

## Abstract

Solitary plasmacytoma is a rare malignant tumor belonging to the family of plasma cell proliferation. It accounts for a small portion of plasma cell tumors and remains a rare condition. We report three cases of rare extraosseous plasmacytomas in young patients. Case 1 describes a 55-year-old patient with multiple cutaneous plasmacytomas who underwent a watchful waiting approach. Case 2 discusses a 44-year-old patient with nasal plasmacytoma who was successfully treated with exclusive radiotherapy. Case 3 reports on a 52-year-old patient with a solitary ovarian plasmacytoma who underwent surgical resection and did not receive adjuvant treatment due to complete resection. The patients did not have multiple myeloma and had different treatment approaches based on the location and extent of the plasmacytoma. Local recurrence and progression to multiple myeloma may occur, justifying prolonged and rigorous monitoring.

## Introduction

Solitary plasmacytoma is a rare malignant tumor of plasma cells, which are key cells in the immune system, characterized by isolated proliferation without signs of dissemination. It represents 3% to 5% of all plasma cell tumors, and its incidence is 0.04 cases per 100,000 individuals [1]. We distinguish solitary bone plasmacytoma (SBP), the most common form, and solitary extraosseous or extramedullary plasmacytomas (EMP), which are much rarer, with 80% localized in the upper respiratory tract [2]. While most plasmacytomas remain localized, some cases may progress to multiple myeloma (MM), highlighting the importance of early detection. We report the observations of three cases of EMP in rare locations - multiple skin lesions, nasal cavity, and ovary - in young patients, illustrating the clinical diversity and rarity of these tumors.

## Case presentation

Case 1

**Figure 1:**
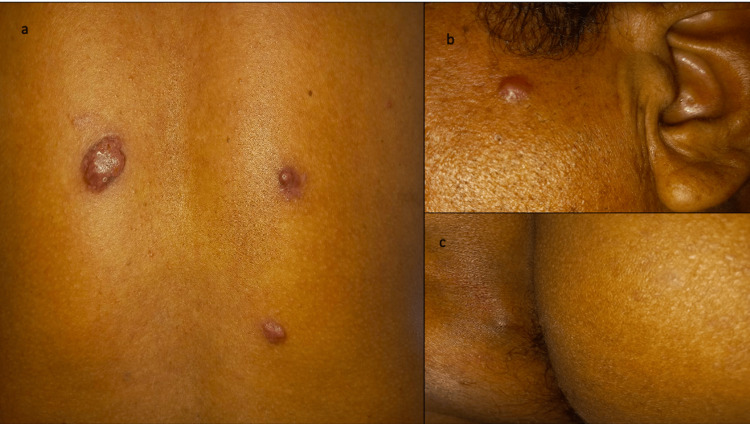
Erythematous papulo-nodular lesions on the back, face, and intergluteal fold (a, b, and c).

A 55-year-old patient with no pathological history presented with multiple cutaneous lesions that had been evolving for seven months in the absence of fever and with preserved general condition. Physical examination revealed a performance status (ECOG) of 0, and dermatological examination showed seven erythematous papulo-nodular lesions, ranging from 0.5 to 2 cm in size, located on the face, right shoulder, right flank, back, and intergluteal fold. The largest lesion, measuring 2 x 2 cm, was located on the dorsal region and had a stable evolution (Figure 1). The rest of the clinical examination was unremarkable. Histological and immunohistochemical analysis of a skin biopsy revealed an inflammatory rearrangement of the skin with the presence of a dense lymphoplasmacytic population that marked the anti-CD138 antibody with a KI67 index of 40%, consistent with a diagnosis of cutaneous plasmacytoma. Further investigations confirmed the primary nature of multiple cutaneous plasmacytoma (MCP). Fluorescence in situ hybridization (FISH) analysis to detect t(4:14), t(14:16), and del 17p was negative. Due to the stability of the skin lesions, their painless nature, the absence of associated MM, and cytogenetic abnormalities suggesting an unfavorable prognosis, as well as the patient's decision, the decision of the multidisciplinary consultation meeting (MCM) was therapeutic abstention, with regular clinical and biological monitoring every three to six months, unless complications or disease progression occur.

Case 2

**Figure 2:**
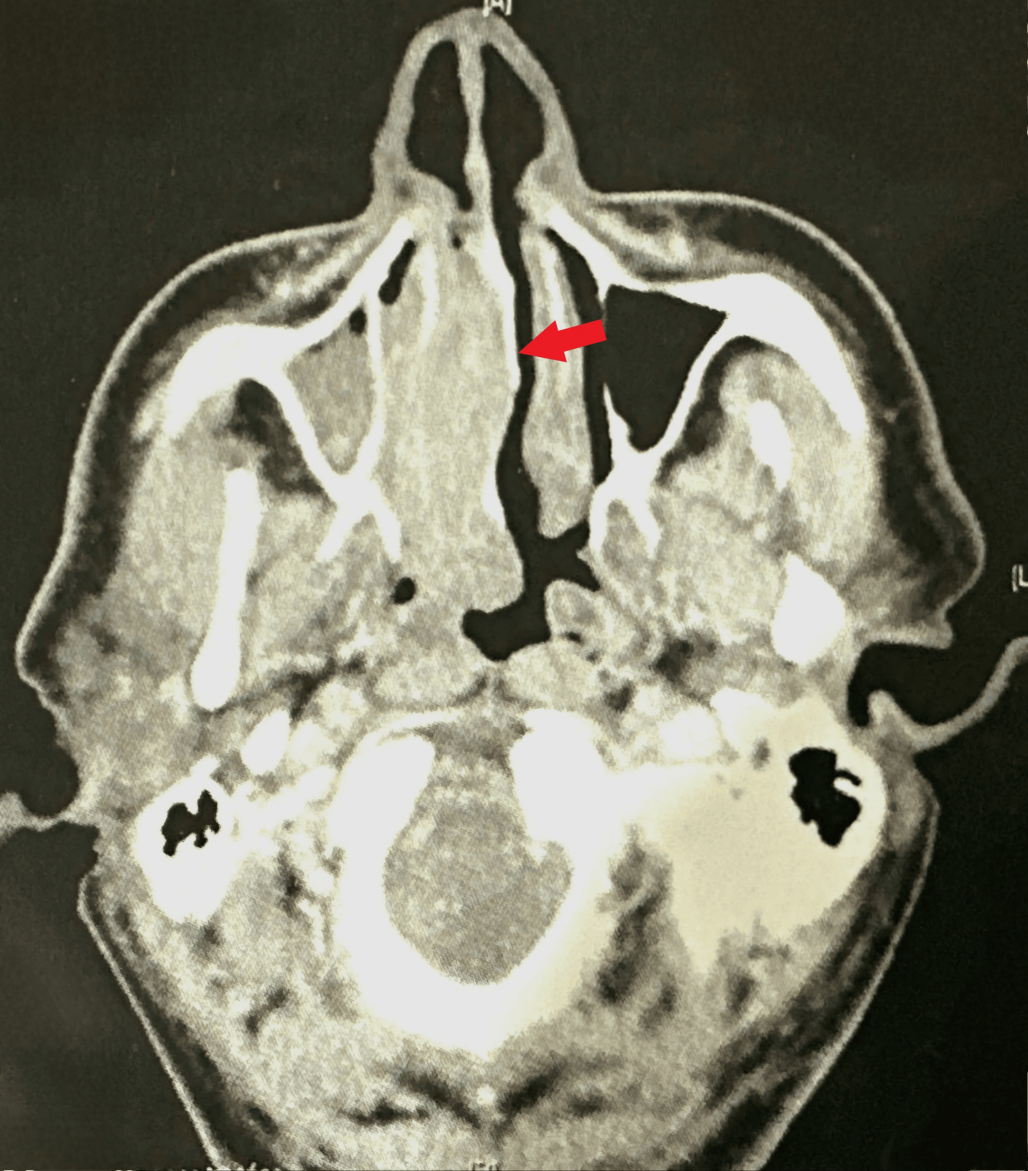
CT scan of the facial mass in the parenchymal window axial section without contrast injection: tumor process in the right antro-choanal fossa measuring (6 x 5 x 2.7 cm), with bone lysis of the nasal septum.

A 44-year-old patient with a history of direct occupational exposure to pesticides for four years. He presented 45 days before his admission, a progressive right nasal obstruction, purulent rhinorrhea, and episodes of epistaxis, all evolving in a context of conservation of the general state. Clinical examination revealed an erythematous mass of firm consistency, occupying the right nasal cavity. A CT scan revealed a homogeneous tissue process measuring 6 x 1.7 x 5 cm in the posterior ¾ of the right nasal cavity (Figure 2). An anatomopathological study of the biopsy revealed cells with plasmocytic differentiation, intensely and diffusely expressing the anti-CD138 antibody. An extension workup excluded associated multiple myeloma (MM), and the diagnosis of right nasal fossa EMP was accepted. The decision of the MCM was to treat the patient with exclusive radiotherapy with a total dose of 50 Gy. At 14 months from the end of radiotherapy, the patient is still in complete remission.

Case 3

**Figure 3:**
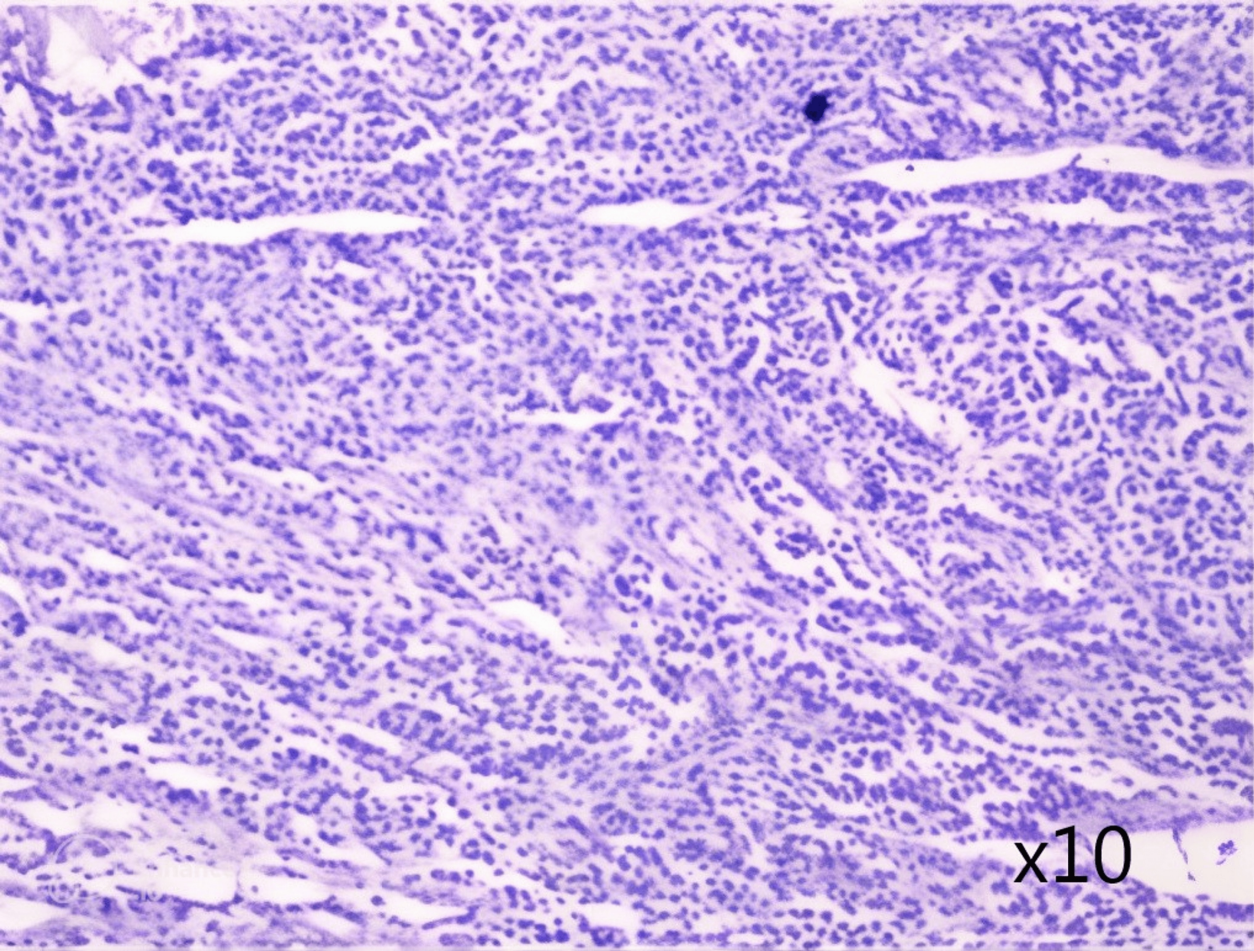
Low power view showing plasma cells arranged in sheets (H & E). H & E: hematoxylin and eosin

**Figure 4:**
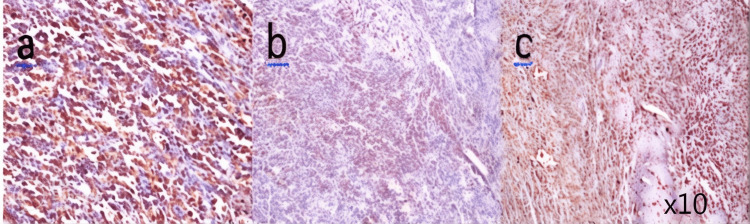
Immunohistochemical staining showing positive expression of epithelial membrane antigen (a), CD138 positive (b), and lambda light chain positive (c).

A 52-year-old multiparous woman, treated for type 2 diabetes under oral treatment for seven years and spontaneous menopause at 49 years without hormonal therapy, presented with recurrent episodes of metrorrhagia for three years. Physical examination showed a stable patient with no signs of hemodynamic instability. A vaginal examination combined with a digital rectal examination showed bilateral filling of the vaginal sac. Pelvic ultrasound revealed an enlarged uterus with heterogeneous endometrial thickening and a heterogeneous left latero-uterine mass. The serum cancer antigen (CA-125) was <4 U/ml and lactic dehydrogenase (LDH) was high at 489 U/l. Beta human chorionic gonadotropin (β-hcg), carcinoembryonic antigen (CEA), and alpha-fetoprotein (AFP) levels were normal. Serum levels of anti-HIV and anti-HbS Ag were also normal. Exploratory laparotomy revealed an enlarged and dilated left ovary measuring 4.5 x 3.5 x 3.0 cm. The endometrium was polypoid with hemorrhagic stigma and an interstitial myoma measuring 0.1 x 0.8 cm in favor of a uterine leiomyoma. Histological study of the left ovary showed a well-circumscribed, encapsulated, white tumor measuring 4.0 x 3.2 cm. Pathological examination revealed tumor proliferation with trabecular and insular architecture with monotonous mature plasma cells and minimal cytonuclear atypia effacing the ovary (Figure 3). Immunohistochemical complement showed positive expression of epithelial membrane antigen (EMA) (Figure. 4a), CD138 (Figure 4b), with lambda light chain restriction (Figure 4c). Ki-67 level was at 10%. Postoperatively, the patient underwent a full workup to exclude MM. Serum protein electrophoresis revealed a small monoclonal protein of 0.4 g/dl present in the gamma region. The diagnosis of solitary ovarian plasmacytoma was retained, and the patient did not receive adjuvant treatment as the surgical resection was complete. The current follow-up is 34 months, and the patient is in good general condition and has no general signs of normalization of her serum monoclonal protein.

## Discussion

EMP is a rare entity characterized by the proliferation of monoclonal plasma cells in the bone marrow, with no evidence of systemic disease other than monoclonal gammopathy that may be present [2,3]. EMP usually affects middle-aged people (median: 55-60 years), particularly women. The risk factors remain unknown; however, previous exposure to radiation has been suggested. EMPs can affect any organ or location; they are most commonly located in the head and neck, accounting for about 80% of cases [1,2].

Primary cutaneous plasmacytoma (PCP) is a rare form of cutaneous B-cell lymphoma, consisting of a malignant monoclonal proliferation of plasma cells, only cutaneous and not associated with MM. It accounts for only 4% of EMPs. The average age is about 60 years, with a male-to-female ratio of 4:1. Solitary forms (60%) are more common than multiple forms (40%) [4]. Clinically, PCP presents as papulonodular of variable size, often 1-5 cm, firm, hemispherical, or lobulated, with varying shades of red, pink-purple, brown, purple, or ecchymosis. They may also have the color of normal skin and rarely ulcerate [5]. The lesions are often asymptomatic but may be painful or pruritic [4,6]. PCP most commonly affects the face, trunk, and limb, especially the extremities, with occasional associated mucosal lesions, especially in the gingiva or labial mucosa. The diagnosis is confirmed by a cutaneous biopsy for anatomopathological study, showing mature, monomorphic plasma cell proliferation with rare cytonuclear atypia, located in the middle dermis, possibly extending to the hypodermis, while sparing the superficial dermis and epidermis. Immunohistochemistry shows expression of CD138, CD79, and CD38, but not CD20 or CD19 [7]. The presence of amyloid deposits should suggest a secondary form [8]. Treatment of multiple or irresectable forms is not standardized, with chemotherapy regimens used for myeloma sometimes being proposed, with or without radiotherapy. Surveillance with therapeutic abstention has been proposed for some asymptomatic patients with stable lesion progression [4]. PCP has a better prognosis than secondary forms [9]. Multiple forms are more aggressive and have higher mortality levels, with a two-year survival rate of no more than 25% [10]. The major prognostic criterion is the tumor burden, represented by the size of the lesions [11].

Nasosinus plasmacytoma is the most common site for EMP [3]. Clinical symptomatology is nonspecific and depends on the stage of tumor progression [12]. CT scans allow localization of the tumor mass and assessment of bone lysis. The diagnosis is histological and immunohistochemical [13]. Radiotherapy is the gold standard treatment for upper aerodigestive tract EMP and permits local control in 70-100% of cases [14]. The recommended dose is between 45 Gy and 50 Gy (for large tumors) [3,14]. The predictive factors for local recurrence appear to be the dose and the accessibility of the tumor site to radiotherapy [15]. Prognostic factors for survival are mainly related to progression to multiple myeloma [1,15].

EMP of the female genital tract is extremely rare, and solitary ovarian plasmacytoma is exceptional [16-19]. These tumors are usually large, exceeding 12 cm, with abdominal pain and/or a mass [18]. Our patient was asymptomatic [20]. The metrorrhagia could be explained by the leiomyoma rather than by the ovarian mass. Preoperatively, an ovarian granulosa tumor was suspected because of the frequency, age, postmenopausal status, and location. The diagnosis requires a macroscopic examination and immunohistochemical study. Ovarian plasmacytomas are more likely to involve the left ovary and are usually without evidence of disseminated disease [18,19]. Adjuvant radiotherapy is proposed when complete resection of the lesion is not possible [2]. In this case, the patient had an asymptomatic left ovarian plasmacytoma and benefited from complete surgical resection without adjuvant radiotherapy.

## Conclusions

EMPs are rare and heterogeneous tumors that can affect any organ, making their clinical diagnosis difficult, so histological and immunohistochemical studies, particularly the detection of CD138, are essential for confirming the diagnosis and differentiating EMPs from other conditions.

Treatment strategies are adapted to the individual case and can range from simple surveillance for stable lesions to systemic chemotherapy and, in some cases, surgical resection or radiotherapy. Notably, local recurrence and progression to MM can occur, justifying prolonged and rigorous monitoring to detect disease evolution and guide timely intervention.
